# Typical and Local Diagnostic Reference Levels for Chest and Abdomen Radiography Examinations in Dubai Health Sector

**DOI:** 10.3390/jimaging11010021

**Published:** 2025-01-13

**Authors:** Entesar Z. Dalah, Maitha M. Al Zarooni, Faryal Y. Binismail, Hashim A. Beevi, Mohammed Siraj, Subrahmanian Pottybindu

**Affiliations:** 1 Central Diagnostic Imaging Department, Dubai Health, Dubai P.O. Box 2727, United Arab Emirates; 2 College of Medicine, Mohammed Bin Rashid University, Dubai Health, Dubai P.O. Box 2727, United Arab Emirates; 3 Diagnostic Imaging Department, Rashid Hospital, Dubai Health, Dubai P.O. Box 2727, United Arab Emirates; maalzarooni@dubaihealth.ae; 4 Diagnostic Imaging Department, Dubai Hospital, Dubai Health, Dubai P.O. Box 2727, United Arab Emirates; fybinismail@dubaihealth.ae; 5 Diagnostic Imaging Department, Hatta Hospital, Dubai Health, Dubai P.O. Box 2727, United Arab Emirates; habeevi@dubaihealth.ae; 6 Diagnostic Imaging Department, PHC Hospital, Dubai Health, Dubai P.O. Box 2727, United Arab Emirates; msiraj@dubaihealth.ae; 7 Diagnostic Imaging Department, Latifa Hospital, Dubai Health, Dubai P.O. Box 2727, United Arab Emirates; spottybindu@dubaihealth.ae

**Keywords:** diagnostic reference levels, chest and abdomen radiography, Dubai health sector, United Arab Emirates, projection radiography, dose area product (DAP)

## Abstract

Chest and abdomen radiographs are the most common radiograph examinations conducted in the Dubai Health sector, with both involving exposure to several radiosensitive organs. Diagnostic reference levels (DRLs) are accepted as an effective safety, optimization, and auditing tool in clinical practice. The present work aims to establish a comprehensive projection and weight-based structured DRL system that allows one to confidently highlight healthcare centers in need of urgent action. The data of a total of 5474 adult males and non-pregnant females who underwent chest and abdomen radiography examinations in five different healthcare centers were collected and retrospectively analyzed. The typical DRL (TDRL) for each healthcare center was established and defined per projection (chest: posterior–anterior (PA), anterior–posterior (AP) and lateral (LAT); abdomen: erect and supine) for a weight band (60–80 kg) and for the whole data (no weight band). Local DRL (LDRL) values were established per project for the selected radiograph for the whole data (no weight band) and the 60–80 kg population. Chest radiography data from 1755 (60–80 kg) images were used to build this comprehensive DRL system (PA: 1471, AP: 252, and LAT: 32). Similarly, 611 (60–80 kg) abdomen radiographs were used to establish a DRL system (erect: 286 and supine: 325). The LDRL values defined per chest and abdomen projection for the weight band group (60–80 kg) were as follows: chest—0.51 PA, 2.46 AP, and 2.13 LAT dGy·cm^2^; abdomen—8.08 for erect and 5.95 for supine dGy·cm^2^. The LDRL defined per abdomen projection for the 60–80 kg weight band highlighted at least one healthcare center in need of optimization. Such a system is efficient, easy to use, and very effective clinically.

## 1. Introduction

Patient radiation safety in the field of diagnostic imaging is a priority that cannot be overlooked or overemphasized. The as low as reasonably achievable (ALARA) principle aims to uphold diagnostically adequate imaging quality while keeping the dose per radiological procedure as low as reasonably achievable. Given the major drawback of radiation-induced risk that is associated with all radiological examinations using X-rays, radiation exposure received during medical examinations should be justified and optimized to ensure that the benefits outweigh the risks [1] and adhere to the ALARA principle.

The concept of the diagnostic reference level (DRL) was introduced and accepted as a radiation safety optimization tool in the field of diagnostic and interventional medical imaging [2,3,4,5,6]. Clinically, DRLs are an effective approach for optimizing patient radiological exposure [7,8,9]. DRLs can be used for internal auditing and to detect abnormally high and low doses across centers and regions [3], thereby indirectly protecting individuals from radiation-induced risks [10,11]. As per an International Commission on Radiation Protection (ICRP) report 135 [3], two-dose quantities are recommended to establish DRLs for projection radiography: the entrance surface dose (ESD) and dose area product (DAP). At present, almost all projection radiography devices are equipped with a DAP meter.

This patient dose report is the second review of the Dubai Health sector, while our first health sector review was conducted in 2020. Recently, we implemented the addition of a 0.1 mm copper (Cu) filter as a standard practice in our common radiography examinations for adult males and non-pregnant females. We achieved this following the pilot study carried out by members of our health sector [12], demonstrating a significant DAP reduction, with no compromise of image quality when additional beam filtering of 0.1 mm copper (Cu) was used.

Here, we report a comprehensive DRL system to optimize radiation exposure of patients undergoing chest and abdomen radiographic examinations within the Dubai health sector. Given that such services are provided by several healthcare centers operating within our health sector, we provide both the typical DRL (TDRL) values and the local DRL (LDRL) values. With optimization as the main objective, TDRLs and LDRLs are established for common projections in the chest and abdomen radiographic examinations performed within the practice. A demonstration of the impact of adding the 0.1 mm Cu filter on the TDRL and LDRL values is considered. Furthermore, dose variation across vendors is demonstrated.

## 2. Materials and Methods

The patient dose database used in this health sector report was approved by our institutional scientific research ethics committee. Patient dose data were collected retrospectively from 1 January to 1 August 2024. The data were collected from a total of five healthcare centers that operate within the Dubai health sector (denoted A, B, C, D, and E). Radiographic devices per room (denoted R) from different vendors and with different detector characteristics were enrolled. Only image receptors using digital radiography detectors (denoted DR) and computed radiography detectors (denoted CR) are used throughout our health sector.

### 2.1. Obtaining the Data

An electronic platform, DOSE TQM version 19.11 (Qaelum NV, Belgium) [13], was used to automatically collect patient dose data. The electronic dose platform is linked with our health sector picture archiving and communication system (PACS). The dose quantity used in this report is DAP measured in dGy·cm^2^. Patient age, weight, projection information, and acquisition parameters, including peak kilovoltage (kVp), tube current time (mAs), source image distance (SID, measured in cm), scan mode (automatic exposure control, AEC or manual), grid ratio, total beam filtration and chamber configuration were all retrieved using the same electronic platform. Using the device tap on the DOST TQM platform allows data retrieval from each device (e.g., radiograph, mammogram, dental, computed tomography) at every healthcare center. Once the radiograph device of interest is selected, data from that device can be exported in Excel format. All the input variables mentioned above can be retrieved for every single patient. The only identification enabled is the study accession number to maintain patient privacy. For every radiography unit enrolled in this study, all patient demographic data, dose information, radiological examination name and protocol, and scan acquisition parameters can be exported into a single Excel sheet.

Here, the DRLs reported are for adult males and non-pregnant females. Furthermore, the DRLs reported are only for fixed non-portable radiograph devices.

### 2.2. DRL Calculation

The TDRL value per healthcare center, despite the number of radiography units (rooms), is represented by the median of the DAP distribution per examination per projection. The LDRL value for the entire Dubai health sector is set at the 3rd quartile (75th percentile) of the TDRL values for all the participating healthcare centers.

For optimization and effective auditing purposes, the TDRL and LDRL for chest radiography were classified based on the posterior–anterior (PA) projection, anterior–posterior (AP) projection, and lateral (LAT) projection. For abdomen radiography, the TDRL and LDRL were classified based on erect and supine projections. Further, a vendor-based comparison is provided involving all of GE Healthcare’s (denoted as GE), Siemens’ (denoted as SIE), Philips’ (denoted as PHI), and Fuji’s (denoted as FUJI) radiography. The TDRL and LDRL values are reported based on classified weight (60–80 kg) and non-classified (whole sample) populations.

**Figure 1:**
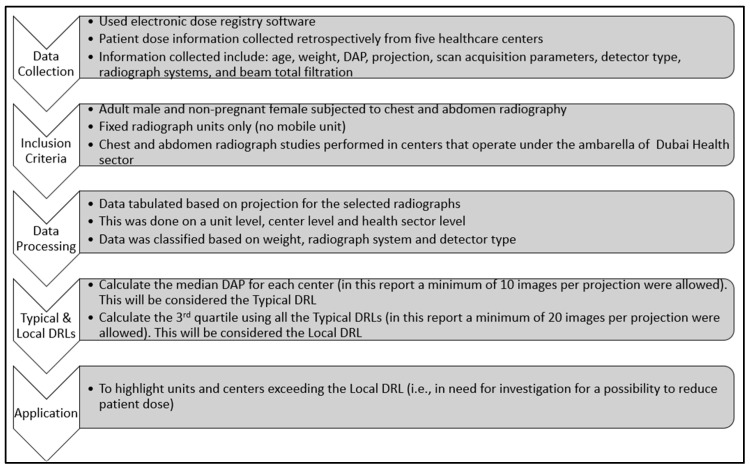
Diagram showing the workflow used to establish the TDRL and LDRL values starting with data collection, covering the inclusion criteria, data processing, setting the TDRL and LDRL values, and concluding with application.

A minimum of 10 cases per image projection for each radiography examination were considered and used to calculate the TDRL values. No TDRL value is reported for projections with fewer than 10 images. The rationale behind the enrollment of 10 images per projection is that the statistical uncertainty among a weight- and projection-restricted cohort of patients is minimal. The TDRL values obtained for each image projection were used to calculate the LDRL values for each image projection. Ideally, a minimum of two healthcare centers are needed to establish the LDRL values. However, LDRLs were considered based on one center, provided that the number of images per projection was ≥20. In this situation, the median of the entire DAP distribution of this single center is calculated to estimate the LDRL. Figure 1 summarizes the workflow used to establish this patient dose report.

### 2.3. Statistical Analysis

Statistical analysis was conducted using GraphPad Prism 8, V8.03, GraphPad Software, La Jolla, CA, USA. Quantitative variables are expressed as the median, minimum (Min), maximum (Max), and 25th and 75th percentiles.

## 3. Results

### 3.1. Database

All five healthcare centers (A to E) operating within the Dubai health sector participated in this patient dose review. The total number of radiography rooms enrolled was 21 (A-R1 to E-R8). Center A has seven rooms, center B has four rooms, center C has one room, center D has one room, and center E has eight rooms.

Out of the 21 radiography units, 20 units were equipped with cesium iodide scintillators coupled to a thin-film transistor (TFT) matrix with amorphous silicon (also known as DR detectors), and 1 unit had a single-panel (non-tiled) amorphous silicon detector with a cesium iodide scintillator (also known as a CR detector).

A total of 5474 and 2366 DAP values for chest and abdomen radiographs were reviewed. The former represents the entire chest and abdomen data (no weight band) and the latter represents the weight band data (60–80 kg) population. The total number of chest radiographs was 4248 (no weight band) and 1755 (60–80 kg). The population per chest radiograph projections was 3681 PA (no weight band) and 1471 (60–80 kg), 506 AP (no weight band) and 252 (60–80 kg), and 61 LAT (no weight band) and 32 (60–80 kg). The total number of abdomen (erect and supine) radiographs was 1226 (no weight band) and 611 (60–80 kg). The populations per abdomen erect projection numbered 591 (no weight band) and 286 (60–80 kg), and per abdomen supine projection, they numbered 635 (no weight band) and 325 (60–80 kg).

The gender-based age distribution per radiograph projection was chest PA (15–102)-year-old male and (15–95)-year-old female, chest AP (15–98)-year-old male and (21–95)-year-old female, chest LAT (20–83)-year-old male and (29–82)-year-old female, abdomen erect (15–95)-year-old male and (15–94)-year-old female, and abdomen supine (15–95)-year-old male and (15–94)-year-old female.

### 3.2. Scan Acquisition Parameters

Table 1 shows the number and distribution of radiography rooms (denoted in numerical series, R1 to R21) across our healthcare centers (A to E), and the scan acquisition parameters used to perform chest X-rays for each PA, AP, and LAT projection.

Table 2 shows the number and distribution of radiography rooms across our healthcare centers (A to E), and the scan acquisition parameters used to perform abdomen X-rays for each erect and supine projection.

### 3.3. TDRL and LDRL Values

**Figure 2:**
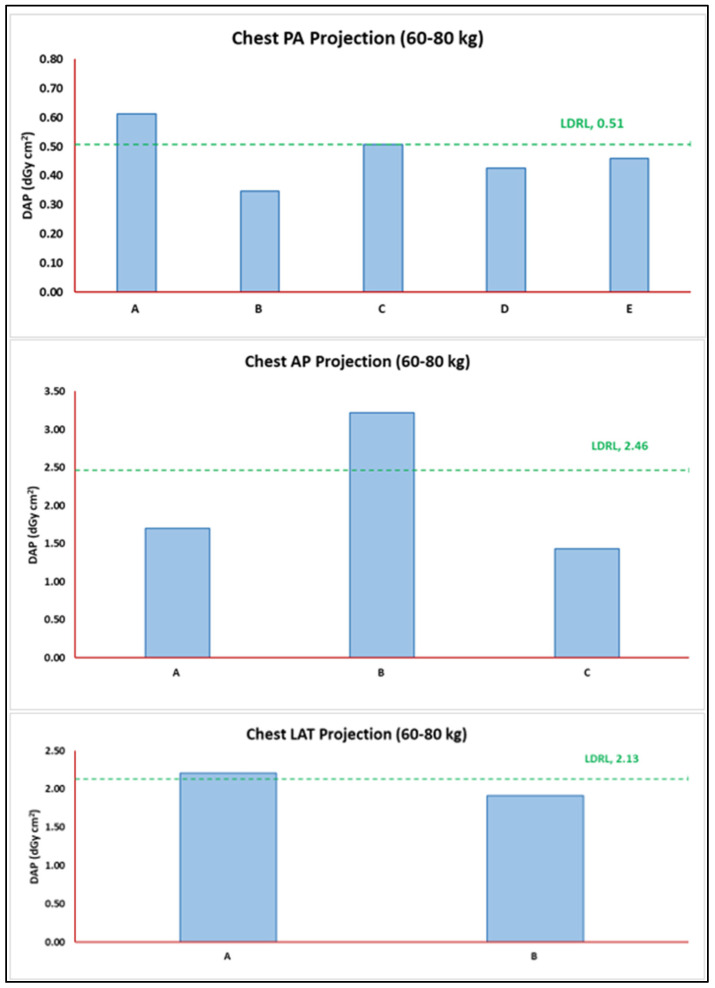
TDRL values for each healthcare center plotted against the obtained LDRL for chest radiograph per projection. Used as an aiding tool for optimization, for example, healthcare centers A, B, and A in PA, AP, and LAT projections, respectively.

**Figure 3:**
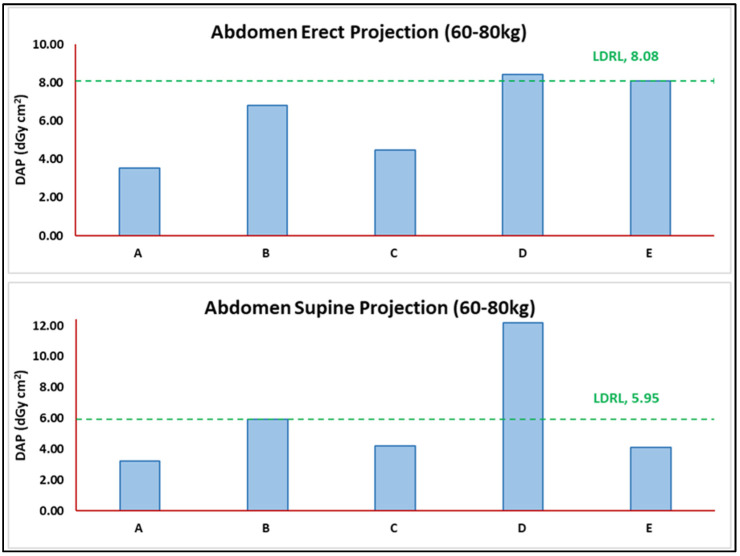
TDRL values for each healthcare center plotted against the obtained LDRL for the abdomen radiograph per projection. Used as an aiding tool for optimization, for example, healthcare center D in erect and supine projections.

The health sector patient TDRL values were based on two decimal medians for the DAP distribution observed for each healthcare center despite the number of radiograph rooms and vendors. The LDRL values were based on two-decimal third quartile (75th percentile) values of all TDRL values calculated for each healthcare center (A to E). Table 3 shows the TDRL distribution values for each healthcare center, the number of rooms, and the number of projections with and without weight bands. The values of the DAP spectrum (distribution) in Table 3 are presented in the form of the 25th percentile, median (TDRLs), and 75th percentile. Figure 2 illustrates the distribution of chest radiograph TDRL (60–80 kg) weight band values against the obtained LDRL value for the specific weight band population per chest radiograph based on projections. Figure 3 shows the distribution of abdomen radiograph TDRL (60–80 kg) weight band values against the obtained LDRL value for the specific weight band population per abdomen radiograph based on projections.

Table 4 presents the LDRL distribution values for the number of centers enrolled, rooms, and projections with and without weight bands for the selected radiograph examinations. The LDRL spectrum in Table 4 is presented in the form of the 25th percentile, median, and 75th percentile (LDRL). Table 5 provides a comparison of the LDRLs established in this patient dose review against some national and international existing DRLs for the same projection radiograph.

**Figure 4:**
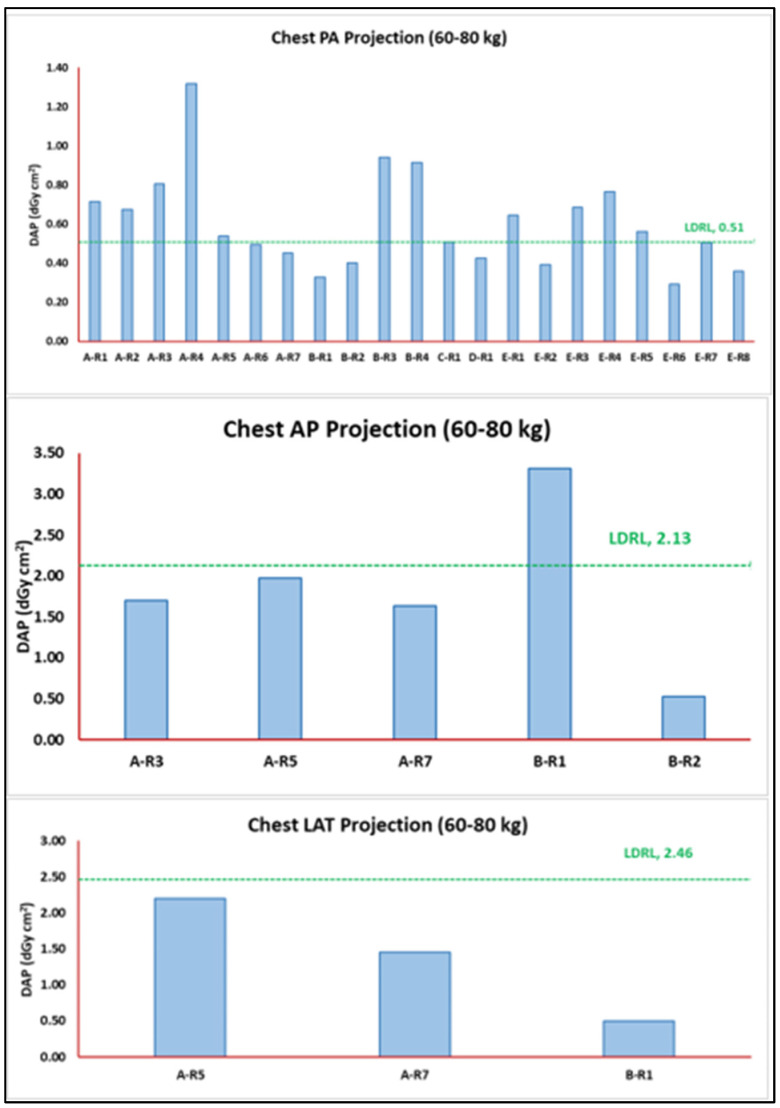
Median DAP values for each room used for the specific chest radiograph plotted against the obtained LDRL for chest radiograph per projection. Used as an aiding tool for optimization; examples of rooms exceeding the LDRLs, B R1 in AP chest projection and rooms A R1 to R5, B R3 and R4 and E R1, R3 to R5 in the PA projection.

The median DAP value for each device was calculated to provide the DAP distribution per room. Figure 4 presents the median DAP per room for PA, AP, and LAT chest radiographs for the weight band population (60–80 kg).

### 3.4. Impact of Additional Beam Filter

**Figure 5:**
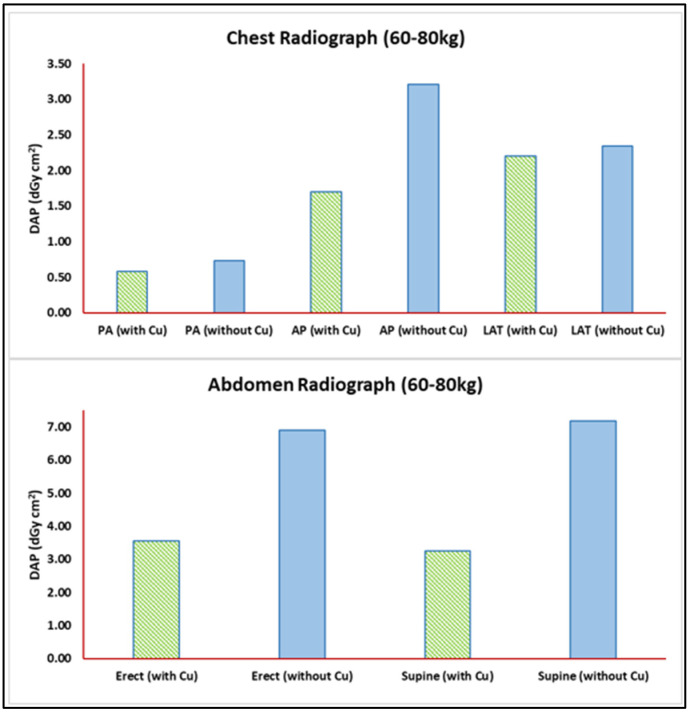
TDRL reduction due to the additional beam filter of 0.1 mm Cu per radiograph projection for weight band population (60–80 kg). Top panel: chest radiograph DAP with and without additional beam filter. Bottom panel: abdomen radiograph DAP with and without additional beam filter.

A 21% DAP TDRL reduction was observed for the chest PA projection as a result of applying the additional 0.1 mm Cu filter. A reduction of more than 60% in DAP TDRL was achieved in the AP projection, and 6% in the LAT projection, as shown in Figure 5 (top panel). A 40% overall DAP LDRL reduction was achieved for the most common chest PA projection. Figure 5 (bottom panel) illustrates the DAP TDRL reduction that was observed for abdomen radiograph examinations, with 64% and 74% reductions in erect and supine projections, respectively.

### 3.5. Vendor DAP Distribution

**Figure 6:**
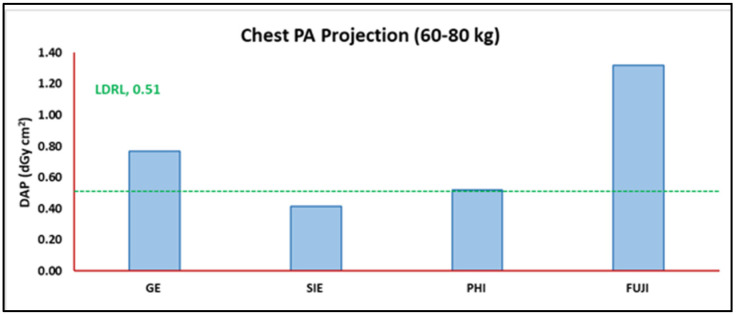
Median DAP values per each vendor for the specific PA chest radiograph plotted against the obtained LDRL weight band PA chest radiograph projection.

Figure 6 shows the DAP distribution observed in PA chest radiographs for the weight (60–80 kg) population. Table 6 provides a descriptive summary based on the specific radiograph, number of rooms, and number of projections for the weight band (60–80 kg) population.

## 4. Discussion

Establishing DRLs is a cornerstone in ensuring the highest standard of care. DRLs serve as a practical tool for achieving three fundamental objectives: patient safety, dose optimization, and internal auditing. This involves a balance between image quality and radiation dose, ensuring that the imaging procedure delivers the maximum diagnostic benefit with the least possible risk. This health sector patient DRL report was made by collecting patients’ measured radiation exposure while undergoing radiographic examination in healthcare centers that operate within the Dubai health sector. Chest and abdomen radiographs are the most common radiograph examinations in our healthcare sector, both involving the exposure of several radiosensitive organs. Several advancements were achieved in the present health sector patient dose report over our first review conducted in 2020. First, we managed to establish the TDRLs and LDRLs based on weight, although the TDRLs and LDRLs reported here are made using a sufficient number of projections, i.e., weight restriction can be waived as per the ICRP report 135 [3]. Reporting typical and local DRLs based on the recommended weight band (60–80 kg) from the ICRP report 135 [3] not only allows for better comparisons against national and international existing DRLs but also allows us to correctly compare against the different radiograph projections for the selected studies. Further, we were able to report the TDRLs and LDRLs per projection, particularly for abdomen radiographs, where different DRL values were proposed for erect and supine abdomen positions separately, as shown in Table 3 and Table 4. Finally, we marked a substantial overall dose reduction in the TDRLs and LDRLs for both chest and abdomen radiographs due to implementing an additional 0.1 mm Cu filter, described in Section 3.4. This intervention (adding 0.1 mm Cu) was made to address a finding in our 2020 review.

DRLs for a chest radiograph differ according to the projection. Herein, the chest AP projection demonstrated the highest DRL value compared to PA and LAT for the same weight band group (60–80 kg). Similarly, the DRLs for an erect abdomen radiograph were higher than a supine abdomen radiograph for the same weight band group (60–80 kg). Our observation is in line with the existing literature summarized in Table 5 for both chest and abdomen radiographs.

Clinical settings (Table 1 and Table 2) for the selected radiographs and the different systems enrolled (Table 6) resulted in notably different DAP values. The SIE radiograph system showed the lowest DAP value, followed by PHI, GE, and Fuji (Figure 6), when performing a PA chest radiography for standard-size patients (60–80 kg). Similarly, Precht et al. [22] observed significant variation in the reported DAP values across different radiograph systems, even across those with the same combinations of kV and mAs. This variation was thought to be partly due to the difference in systems’ beam filtration and because some systems’ kV and mAs combinations were not available. However, Precht et al. [22] were not able to explain the significantly different DAP values reported from Canon and Siemens systems used in their study, with both systems having the same beam spectral shape (i.e., the same filter system). They concluded that the evident variations witnessed in their reported DAP values were actually attributable to the differences in the X-ray tube generator system characteristics and specifications. Sundell et al. [23], who studied mammogram doses in different mammography systems, reached the same conclusions. Tube generator system differences may result in unequal image quality adequacy [22,23].

Regarding the impact of detector systems on DAP, Joregensen et al. [24] conducted a study to compare the radiation dose and image quality using two different radiograph detector systems, DR and CR. They concluded that the DR system offers a considerable dose reduction compared to the CR system, with no issues with image quality. This has been attributed to the advancements made in the DR detector systems, which are more efficient and have high X-ray beam quality, given the additional beam tube filtration used. Tonkopi et al. [25] reported LDRL values of 0.09 mGy and 1.1 mGy for DR and CR, respectively, for the PA chest radiograph. This considerable dose reduction promotes the need to establish DRLs based on the detector system type.

Evidently, adequate beam tube filtration is instrumental in shaping and strengthening the X-ray beam’s spectral quality [26,27], resulting in a lower radiation dose for the patient. Using different thicknesses of Cu sheets, Siraj and colleagues [12] comprehensively studied the impact of additional beam filtration on different dose metrics including exposure index (EI), ESD, DAP, and image quality. Of the three different Cu filter thicknesses used (0.1 mm, 0.2 mm, and 0.3 mm), applying an additional filter of 0.1 mm Cu to the primary beam results in significant DAP reduction without compromising the image quality for patients subjected to chest radiography.

In the case where TDRLs exceed the LDRL value or any reported DRL for a selected radiograph study, one needs to list all the possible factors that could contribute to dose variations. Such factors can be used to justify the DRL variations. In association with the discussion above, radiograph systems (SIE, GE, PHI, Fuji, Canon, etc.) can clearly be a confiding factor. In the present work (Figure 6), the SIE radiograph system yielded the lowest DAP value compared to the remaining vendors when performing a PA chest radiograph for standard-size patients (60–80 kg). Hence, when the TDRL (representing a unit or center) exceeds the LDRL (representing a healthcare sector), a radiography system can be used to justify the excessive dose observed. Likewise, detector type (DR or CR) could also explain the TDRL variation alongside different TDRL and LDRL values. In our case (Figure 4, chest PA projection), the DAP values reported in A-R1 and A-R2 (both GE systems with a DR detector) are lower than the DAP value reported in A-R3 (GE system with a CR detector). Although all three units (A-R1, A-R2, and A-R3) are GE systems and come with the same default (2.7 mm Al) and additional beam filtration system (0.1 mm Cu), in this case, the type of detector justifies the excessive variation observed in DAP.

Importantly, the selected scan acquisition parameters play a role in the resulting DAP value, for example, the variation seen in the two SIE systems (A-R6 and A-R7) used to perform PA chest radiography (Figure 4, chest PA projection), with A-R7 yielding a lower DRL value. In this case, both systems come with a DR-type detector and have the same total beam filtration system (3.62 mm Al + 0.1 mm Cu). However, the PA radiographs were acquired with different scan acquisition parameters. While the kVp range for A-R6 was lower than A-R7, (70–125) vs. (125–137), respectively, the mAs range for A-R7 was higher than A-R6, (2.0–90.0) vs. (3.0–46.0), respectively. Further, A-R6 scans started at a closer SID than those of A-R7. The higher-end mAs and closer SID distance both contributed to the higher DRL value seen in A-R7.

As shown in Figure 4, chest AP projection is another valuable example of using such a comprehensive DRL system to outline potential sources of the excessive DRL associated with B-R1 vs. B-R2 and the calculated LDRL. Both B-R1 (exceeding the LDRL value for chest AP projection for standard-size patients, 60-80 kg) and B-R2 (lowest TDRL value reported for the same projection and weight group) are SIE systems that are equipped with a DR-type detector. The total filtration system associated with the unit exceeding the LDRL is 1 mm Al + 0.1 mm Cu, whereas the total filtration system associated with the unit yielding the lowest TDRL (B-R2) is 2.7 mm Al + 0.1 mm Cu. In addition, the mAs range for the unit exceeding the LDRL value is (0.72–4.56) vs. (0.21–2.24) for the unit yielding the lowest TDRL value (B-R2). Evidently, B-R1 (unit exceeding the LDRL value) uses lower beam quality since the default filter is less than 2.5 mm Al. This would result in both a high patient dose (i.e., DAP) and a noisy image. To compensate for the noise, the mAs needs to be at a higher range, which is exactly what is happening in B-R1. In relation to this, the TDRL for B-R1 is high to the point of exceeding the LDRL. For optimization, one would need to explore the possibility of increasing the default filter to a minimum of 2.5 mm Al. To the authors’ knowledge, the present patient-based dose review is the first comprehensive radiography DRL report in the United Arab Emirates (UAE). Few studies have reported abdomen DRLs based on projections, with the majority of these being reported for the entire abdomen [15,16,17,28]. Herein, not only do we report erect and supine abdomen DRLs separately, but we also report DRLs using weight bands and extra beam filtration. The fact that we included only one radiograph unit with a CR detector system eliminated the possibility of conducting a dose comparison between the DR and CR detector systems in this patient review. Further, mobile (portable) X-ray radiograph units and extremity radiographs were not part of this study. This should be addressed in a separate study, given the huge number of variables associated with mobile X-ray radiography, such as inconsistent positioning and scan acquisition parameters. Another potential limitation of this study is the inability to report the actual field size as this important input variable is not reported among the digital image and communication in medicine (DICOM) header details. Similarly, acquisition parameters for some projections were not listed in the DICOM header details. A similar situation and limitation were reported by Alshamrani et al. [14]. Image quality assessment, whether qualitative or quantitative, was not part of this patient dose review report; however, there was no evidence suggesting the need for a repeat study due to compromised quality.

## 5. Conclusions

TDRLs and LDRLs were established for adult males and non-pregnant females subjected to chest and abdomen radiography. The DRLs established in this review were based on the projection and weight band, allowing for an effective auditing review. Dubai Health LDRL values are in line with most of the national and international reported DRLs.

## Figures and Tables

**Table 1:** Room distribution and scan acquisition parameters are used to perform chest radiography across our healthcare centers (A to E).

Room	Projection PA/LAT/AP	kVp Min–Max	mAs Min–Max	SID (cm) Min–Max	Mode AEC/Manual	Grid Ratio	Total Beam Filtration
A-R1 (DR-GE)	PA	120–130	0.71–8.30	180	AEC	6:1	2.7 mm Al (Default) + 0.1 mm Cu (Additional)
	AP	95–100	2.10–11.13	–	AEC	None	
	LAT	110–120	2.10–8.70	180	AEC	6:1	
A-R2 (DR-GE)	PA	95–130	0.72–14.21	180	AEC	6:1	2.7 mm Al (Default) + 0.1 mm Cu (Additional)
	AP	95–100	0.55–1.26	–	AEC	None	
	LAT	100–120	2.43–160.3	180	AEC	6:1	
A-R3 (CR-GE)	PA	95–130	0.72–14.21	180	AEC	6:1	2.7 mm Al (Default) + 0.1 mm Cu (Additional)
	AP	65–142	0.50–19.84	–	AEC	None	
	LAT	110–120	2.39–17.09	180	AEC	6:1	
A-R4 (DR-FUJI)	PA	61–129	1.00–10.00	151–206	AEC	12:1	-
A-R5 (DR-PHI)	PA	70–125	3.00–46.00	180–181	AEC	8:1	3.38 mm Al (Default) + 0.1 mm Cu (Additional)
	AP	70–125	1.51–3.58	–	AEC	-	
	LAT	125	1.88–17.50	110–181	AEC	8:1	
A-R6 (DR-SIE)	PA	70–125	3.00–46.00	180–181	AEC	13:1	3.62 mm Al (Default) + 0.1 mm Cu (Additional)
	AP	85–90	7.00–9.00	116–183	AEC	13:1	
	LAT	125	4.00–46.00	180	AEC	13:1	
A-R7 (DR-SIE)	PA	125–137	2.00–90.00	116–183	AEC	13:1	3.62 mm Al (Default) + 0.1 mm Cu (Additional)
	AP	60–125	2.00–37.00	94–190	AEC	13:1	
	LAT	125	3.00–103.00	167–180	AEC	13:1	
B-R1 (DR-SIE)	PA	60–129	0.50–30.51	107–224	Manual	13:1	1 mm Al (Default) + 0.1 mm Cu (Additional)
	AP	84–125	0.72–4.65	115–180	Manual	13:1	
	LAT	113–129	0.67–66.15	180	Manual	13:1	
B-R2 (DR-SIE)	PA	70–145	0.50–7.20	115–182	-	13:1	2.7 mm Al (Default) + 0.1 mm Cu (Additional)
	AP	68–125	0.21–2.24	115–180	-	13:1	
	LAT	125	0.90–4.84	150–180	-	13:1	
B-R3 (DR-GE)	PA	120	0.78–7.95	180	AEC	6:1	2.7 mm Al (Default) + 0.1 mm Cu (Additional)
B-R4 (DR-GE)	PA	120	0.85–6.36	180	AEC	6:1	2.7 mm Al (Default) + 0.1 mm Cu (Additional)
C-R1 (DR-SIE)	PA	125	0.90–4.40	149–210	AEC	13:1	1 mm Al (Default) + 0.1 mm Cu (Additional)
	AP	85–125	1.70–5.50	107–176	ACE and Manual	13:1	1 mm Al (Default)
D-R1 (DR-PHI)	PA	77–125	1.00–1.70	168–180	AEC	8:1	1 mm Al (Default) + 0.1 mm Cu (Additional)
E-R1 (DR-SIE)	PA	125– 125	0.96– 5.40	180–236	AEC	13:1	2.5 mm Al (Default) + 0.1 mm Cu (Additional)
E-R2 (DR-SIE)	PA	125– 126	0.63– 3.51	180	AEC	13:1	2.5 mm Al (Default) + 0.1 mm Cu (Additional)
E-R3 (DR-GE)	PA	125	0.90–4.28	180	AEC	13:1	2.5 mm Al (Default) + 0.1 mm Cu (Additional)
E-R4 (DR-PHI)	PA	125	1.00–3.10	151–210	AEC	8:1	2.5 mm Al (Default) + 1 mm Al + 0.1 mm Cu (Additional)
E-R5 (DR-PHI)	PA	125	1.20–1.90	151–180	AEC	8:1	2.5 mm Al (Default) + 1 mm Al + 0.1 mm Cu (Additional)
E-R6 (DR-SIE)	PA	125	0.90–4.80	179–180	AEC	13:1	2.5 mm Al (Default) + 0.1 mm Cu (Additional)
E-R7 (DR-PHI)	PA	125	1.00–1.30	178–253	AEC	8:1	2.5 mm Al (Default) + 1 mm Al + 0.1 mm Cu (Additional)
E-R8 (DR-SIE)	PA	125	1.00–9.70	179–180	AEC	13:1	2.5 mm Al (Default) + 0.1 mm Cu (Additional)

kVp (peak tube voltage); mAs (current time); SID (source image distance).

**Table 2:** Room distribution and scan acquisition parameters are used to perform abdomen radiography across our healthcare centers (A to E).

Room	Projection Supine/Erect	kVp Min–Max	mAs Min–Max	SID (cm) Min–Max	Mode AEC/Manual	Grid Ratio	Total Beam Filtration	Chamber Configuration
A-R6 (DR-SIE)	Erect	83	2.60–43.90	115	AEC	13:1	3.62 mm Al (Default) + 0.1 mm Cu (Additional)	Lateral
	Supine	83	6.80–140.30	115	AEC	13:1		Medial and Lateral
A-R7 (DR-SIE)	Erect	81–117	1.80–249.80	115–195	AEC	13:1	3.62 mm Al (Default) + 0.1 mm Cu (Additional)	Lateral
	Supine	81–87	2.40–93.00	91–115	AEC	13:1		Medial and Lateral
B-R1 (DR-SIE)	Supine	80–90	2.79–63.12	98–180	Manual	13:1	1 mm Al (Default) + 0.1 mm Cu (Additional)	Medial and Lateral
	Erect	81–91	8.42–68.48	107–202	Manual	13:1		Lateral
B-R2 (DR-SIE)	Supine	81	11.93–25.89	115–146	Manual	13:1	2.7 mm Al (Default) + 0.1 mm Cu (Additional)	Medial and Lateral
	Erect	82	73.37	180	Manual	13:1		Lateral
C-R1 (DR-SIE)	Erect	75–93	2.00–155.7.0	113–176	AEC	13:1	1 mm Al (Default) + 0.1 mm Cu (Additional)	Lateral
	Supine	81–96	1.70–74.50	113–115	AEC	13:1		Medial and Lateral
D-R1 (DR-PHI)	Erect	70–85	1.40–59.10	115–150	AEC	8:1	1 mm Al (Default) + 0.1 mm Cu (Additional)	Lateral
	Supine	77–96	3.80–30.60	115	AEC	8:1		Lateral and Medial
E-R1 (DR-SIE)	Erect	81– 81	4.66–177.42	106–180	AEC	13:1	2.5 mm Al (Default) + 0.1 mm Cu (Additional)	Lateral
	Supine	81– 81	5.47–35.90	106–150	AEC	13:1		Medial
E-R2 (DR-SIE)	Erect	81	2.81–96.14	102–180	AEC	13:1	2.5 mm Al (Default) + 0.1 mm Cu (Additional)	Lateral
	Supine	81–85	4.85–75.25	105–155	AEC	13:1		Medial

kVp (peak tube voltage); mAs (current time); SID (source image distance).

**Table 3:** Dose area product (DAP) spectrum including typical DRLs and the 25th and 75th percentiles for adult males and non-pregnant females. * refers to centers exceeding the LDRL.

Radiograph	Center	Rooms	Weight (kg)	Number of Projections	DAP (dGy·cm^2^)		
					25th PER	Median (TDRL)	75th PER
CHEST PA	A	7	33–167	1304	0.50	0.67 *	0.92
			60–80	652	0.49	0.61 *	0.80
	B	4	37–175	1574	0.31	0.42	0.69
			60–80	445	0.29	0.35	0.44
	C	1	39–157	436	0.45	0.58	0.73
			60–80	209	0.43	0.51	0.63
	D	1	49–102	38	0.38	0.48	0.67
			60–80	21	0.38	0.43	0.52
	E	8	27–127	329	0.38	0.51	0.73
			60–80	144	0.36	0.46	0.69
CHEST AP	A	3	24–145	370	1.49	1.70	1.75
			60–80	185	1.49	1.70	1.73
	B	2	45–194	119	1.01	3.28 *	4.02
			60–80	56	0.91	3.22 *	3.96
	C	1	54–133	17	1.30	1.44	2.98
			60–80	11	1.33	1.44	2.72
CHEST LAT	A	2	49–126	33	1.27	2.52 *	4.76
			60–80	17	1.33	2.21	3.17
	B	1	50–94	28	0.49	1.31	4.74
			60–80	15	0.49	1.91	5.29
ABDOMEN ERECT	A	2	36–160	205	2.41	3.83	6.33
			60–80	118	2.43	3.55	5.29
	B	1	40–169	215	3.99	7.18	10.98
			60–80	94	4.41	6.80	9.08
	C	1	39–137	110	3.66	7.08	12.20
			60–80	44	3.32	4.50	6.85
	D	1	22–99	16	3.26	8.28 *	15.13
			60–80	11	4.52	8.42 *	19.08
	E	2	45–108	45	4.96	7.34	16.05
			60–80	19	5.81	8.08	12.19
ABDOMEN SUPINE	A	2	36–160	220	2.51	4.05	6.39
			60–80	118	2.57	3.26	4.90
	B	1	38–114	263	3.61	6.20	9.46
			60–80	149	3.97	5.95	8.05
	C	1	30–137	110	3.45	5.31	9.77
			60–80	44	2.86	4.20	5.19
	D	1	45–99	17	11.04	12.30 *	22.07
			60–80	11	11.16	12.20 *	17.87
	E	2	45–108	25	3.34	4.95	7.11
			60–80	13	3.34	4.11	5.16

**Table 4:** Dose area product (DAP) spectrum including local DRLs and the 25th and 75th percentiles for adult males and non-pregnant females.

Radiograph	Number of Centers	Rooms	Weight (kg)	Number of Projections	DAP (dGy·cm^2^)		
					25th PER	Median	75th PER (LDRL)
Chest PA	5	21	27–175	3918	0.48	0.51	0.58
			60–80	1471	0.43	0.46	0.51
Chest AP	3	6	24–194	525	1.57	1.70	2.47
			60–80	252	1.57	1.70	2.46
Chest LAT	2	3	49–126	61	1.61	1.91	2.21
			60–80	32	1.99	2.06	2.13
Abdomen Erect	5	7	22–169	596	7.08	7.18	7.34
			60–80	286	4.50	6.80	8.08
Abdomen Supine	5	7	30–160	647	4.97	5.31	6.20
			60–80	336	4.11	4.20	5.95

**Table 5:** Dose area product (DAP) local DRLs compared to national and international reported DRLs for the same projection radiograph.

Radiograph	Weight (kg)	DRLs DAP (dGy·cm^2^)	
		Present Study	Literature
Chest PA	27–175	0.58	0.88 [14], 1.2 [15], 1.3 [16], 2.5 [17], 3.14 [18], 1.0 [19]
	60–80	0.51	
Chest AP	24–194	2.47	1.3 [15], 1.5 [19], 8.87 [20]
	60–80	2.46	
Chest LAT	49–126	2.21	3.25 [14], 9.07 [20], 4.2 [16], 10 [17]
	60–80	2.13	
Abdomen Erect	22–169	7.34	19.86 [21] **
	60–80	8.08	
Abdomen Supine	30–160	6.20	10.11 [21] **
	60–80	5.95	

** The DAP value is reported in the mean, not the median or 75th percentile.

**Table 6:** Vendor-based dose area product (DAP) spectrum for adults (60–80 kg). * refers to DAP exceeding LDRLs.

Vender	Radiography	Number of Hospitals	Rooms	Number of Projections	DAP (dGy·cm^2^)		
					25th PER	Median	75th PER
SIE	CHEST PA	5	11	926	0.33	0.42	0.55
	CHEST AP	2	5	72	1.05	2.66 *	3.43
	CHEST LAT	2	3	14	0.56	1.13	4.00
	ABDOMEN ERECT	4	6	275	3.18	5.07	7.28
	ABDOMEN SUPINE	4	6	325	2.85	4.53	6.52
GE	CHEST PA	3	7	310	0.64	0.77 *	0.94
	CHEST AP	2	5	173	1.48	1.70	1.72
	CHEST LAT	1	1	2	-	-	-
PHI	CHEST PA	3	5	258	0.44	0.52	0.61
	CHEST AP	2	2	7	-	-	-
	CHEST LAT	1	1	7	-	-	-
	ABDOMEN ERECT	1	1	11	4.52	8.42 *	19.08
	ABDOMEN SUPINE	1	1	11	11.16	12.20 *	17.87
FUJI	CHEST PA	1	1	19	1.10	1.32 *	1.89

## Data Availability

The original contributions presented in this study are included in the article. Further inquiries can be directed to the corresponding author.

## Acronyms

ALARA	As low as reasonably achievable
DRL	Diagnostic reference level
ICRP	International Commission on Radiation Protection
ESD	Entrance surface dose
DAP	Dose area product
Cu	Copper
TDRL	Typical diagnostic reference level
LDRL	Local diagnostic reference level
DR	Digital radiography
CR	Computed radiography
kVp	Peak kilovoltage
mAs	Tube current time
SID	Source image distance
AEC	Automatic exposure control
PA	Posterior–anterior
AP	Anterior–posterior
LAT	Lateral
GE	General electric healthcare
SIE	Siemens
PHI	Philips
R	Room
Min	Minimum
Max	Maximum
AI	Aluminum
EI	Exposure index
UAE	United Arab Emirates
DICOM	Digital image and communication in medicine
PACS	Picture archiving and communication system
